# How Increases in Railway Fares Affect the Devonshire Hospital

**Published:** 1916-12-30

**Authors:** W. Stevenson

**Affiliations:** General Superintendent and Secretary. Buxton, Derbyshire.


*To the Editor of* The Hospital.

### How Increases in Railway Fares affect the Devonshire Hospital.

*To the Editor of* The Hospital.

Sir,—When the revised railway fares come into operation, unless special provision is made for the poor patients who seek health at this national hospital (the largest of its type in the world), I fear the good that is being done here will be greatly curtailed. More than 4,000 poor people have to come to the Devonshire Hospital, Buxton, each year for special treatment for rheumatism and allied diseases from every county in England and Wales, Scotland, and Ireland. They have of necessity to be poor and unable to pay for private treatment in Buxton, or they would not be eligible for admission to this charitable institution. Even as it is the patients find the railway fare a very serious drain upon their resources, and frequently they have to endure severe hardships to get here. It would be a national calamity if this hospital were unable to treat all its applicants, or if they were debarred from its privileges by increased railway fares. Every patient is in possession of an admission card which is sent to them before they come here for treatment, and it would be quite easy to identify them at the railway booking offices. Our chairman, His Grace the Duke of Devonshire, would have lent his support to this endeavour had he been in England. I hope you will kindly use the influence of your paper to enable us to obtain concessions from the various railway companies for our patients. Thanking you in anticipation.—Your obedient servant,

W. Stevenson,

General Superintendent and Secretary.

Buxton, Derbyshire.

